# Immunostimulatory Activity of Synbiotics Using *Lactococcus lactis* SG-030 and Glucooligosaccharides from *Weissella cibaria* YRK005

**DOI:** 10.3390/microorganisms9122437

**Published:** 2021-11-25

**Authors:** Ayeon Kwon, Young-Seo Park

**Affiliations:** Department of Food Science and Biotechnology, Gachon University, Seongnam-si 13120, Korea; kkayeon96@gmail.com

**Keywords:** lactic acid bacteria, *Lactococcus lactis*, *Weissella cibaria*, synbiotics, probiotics, prebiotics, glucooligosaccharides, immunostimulatory activity

## Abstract

Much attention has been recently paid to the health benefits of synbiotics, a combination of probiotics and prebiotics. In this study, synbiotics were prepared by combining lactic acid bacteria with potential as probiotics and purified glucooligosaccharides, and their immunostimulatory activity was evaluated using RAW 264.7 macrophage cells. A lactic acid bacteria strain with high antioxidant activity, acid and bile salt tolerance, adhesion to Caco-2 cells, and nitric oxide (NO) production was selected as a potential probiotic strain. The selected strain, isolated from forsythia, was identified as *Lactococcus lactis* SG-030. The purified glucooligosaccharides produced from *Weissella cibaria* YRK005 were used as prebiotics. RAW 264.7 cells were treated with synbiotics in two ways. One way was a simultaneous treatment with lactic acid bacteria and glucooligosaccharides. The other way was to pre-culture the lactic acid bacteria with glucooligosaccharides followed by treatment with synbiotic culture broth or synbiotic culture supernatant. In both cases, synbiotics synergistically increased NO production in RAW 264.7 cells. In addition, synbiotics treatment increased the expression of tissue necrosis factor-α, interleukin (IL)-1β, IL-6, and inducible nitric oxide synthase genes. Synbiotics also increased the expression of P38, extracellular signal-regulated kinases, c-Jun *N*-terminal kinases, phosphoinositide 3-kinase, and Akt proteins. The results confirmed that the synbiotics prepared in this study exhibited synergistic immunostimulatory activity.

## 1. Introduction

Recently, research on probiotics, prebiotics, and their combination, synbiotics, has been actively conducted along with microbiome research because of their beneficial health functions. In 2001, the FAO and WHO defined probiotics as live microorganisms which confer health benefits on the host when administered in adequate amounts [1]. Probiotics were initially used to improve animal and human health through intestinal microflora control, and today, there is ample evidence of the health benefits of probiotics [2]. Probiotics stimulate and modulate the immune system of the host by activating specific genes in localized host cells [3]. Moreover, health benefits such as anti-inflammatory, antioxidant, and antihypertensive activities have been reported [4].

Prebiotics refers to some non-digestible but fermentable dietary carbohydrates that can selectively stimulate certain bacteria in the colon, such as bifidobacteria and lactobacilli, considered beneficial to humans. Short-chain carbohydrates are also referred to as indigestible oligosaccharides [5]. Prebiotics are defined as non-digestible food ingredients that beneficially affect the host by selectively stimulating the growth and/or activating the metabolism of one or a limited number of health-promoting bacteria in the intestinal tract, thus improving host health [6]. Well-known prebiotics include fructooligosaccharides produced from sucrose, inulin, galactooligosaccharides, lactulose, soy oligosaccharides, etc., and these selectively stimulate selected bacterial strains [7]. Prebiotics offer various benefits to the host, especially on gut health, such as reducing the duration of diarrhea, protective effects to prevent colon cancer, improvement of the gut mucosal barrier, and prevention of inflammatory bowel diseases [8]. In addition, prebiotics stimulate the propagation of beneficial bacteria in the intestine, increasing organic acid, reducing intestinal pH, and growth inhibition of pathogens [9]. Prebiotics can provide the energy needed for the selective species of bacteria responsible for producing short-chain fatty acids (SCFAs). The resulting SCFAs mainly include acetic acid, propionic acid, and butyric acid. These SCFAs have health benefits such as blood lipid modulation, intestinal or systemic immune regulation, and intestinal cell proliferation [10].

Synbiotics are a product consisting of a combination of probiotics and prebiotics and were defined as a mixture of probiotics and prebiotics that beneficially affects the host by improving the survival and implantation of live microbial dietary supplements in the gastrointestinal tract by selectively stimulating the growth and/or activating the metabolism of one or a limited number of health-promoting bacteria, and thus improving host welfare [6]. With recent evidence of the health effects of probiotics and prebiotics, interest in functional foods containing synbiotics has also increased. Many studies showed that when probiotics and prebiotics work together in a living system, the beneficial effects on gut health, prevention, and treatment of disease are synergistically improved [8].

In synbiotic combination, the proper selection of probiotics and prebiotics is an important criterion. Each probiotic and prebiotics should be able to benefit host health even when used separately. Synbiotics likewise have several health effects, such as promoting the growth of probiotic bacteria in the gastrointestinal tract and improving the survival of beneficial microorganisms [11,12]. The health benefits of synbiotics for humans are as follows: (1) Increasing the number of lactobacilli and bifidobacteria and maintaining the balance of the intestinal microflora, (2) Improving immunomodulatory effects, (3) Improving liver function in surgery patients, and (4) Prevention of bacterial translocation [13,14].

Although there is growing interest in and studies on synbiotics, the current studies on synbiotics are limited to using natural carbohydrate sources from plants such as sugar cane fiber or well-established prebiotics such as fructooligosaccharides as prebiotics [15,16]. In the previous study, oligosaccharides from *Weissella cibaria* YRK005 were purified, and their structure was identified to be glucooligosaccharides, one of the emerging prebiotics [17].

In this study, the synbiotics were prepared by combining purified glucooligosaccharides from *Weissella cibaria* YRK005 as prebiotics and potentially probiotic lactic acid bacteria *Lactococcus lactis* SG-030 as probiotics, and their immunostimulatory activity was evaluated by determining the level of nitric oxide production and the expression of genes encoding cytokines such as tissue necrosis factor-α, interleukin (IL)-1β, IL-6, and inducible nitric oxide synthase gene in RAW 264.7 macrophage cells. The expression level of genes involved in the MAPKs signaling pathways and PI3K/Akt signaling pathways such as P38, extracellular signal-regulated kinases, c-Jun *N*-terminal kinases, phosphoinositide 3-kinase, and Akt proteins was also evaluated.

## 2. Materials and Methods

### 2.1. Isolation of Lactic Acid Bacteria Strains and Growth Conditions

Fermented foods such as kimchi and salted fish, seafood, and plant flowers and fruits were collected from several local markets and fields in Korea to isolate lactic acid bacteria. Ten grams of each sample were suspended in sterilized saline (0.88% (*w/v*) NaCl), serially diluted with decimals, plated on PCA agar (5 g tryptone, 2.5 g yeast extract, 1.0 g glucose, and 15.0 g agar, per liter, KisanBio, Seoul, Korea) with 0.005% bromocresol purple, and incubated anaerobically at 37 °C for 18 h. The pure isolated strains by selecting colonies with a different morphology from each plate were anaerobically cultured and maintained on lactobacilli de Man Rogosa Sharpe (MRS; BD, Franklin Lakes, NJ, USA), consisting of 20 g glucose, 10 g peptone, 8 g meat extract, 4 g yeast extract, 5 g sodium acetate trihydrate, 2 g triammonium citrate, 2 g dipotassium hydrogen phosphate, 0.2 g magnesium sulfate heptahydrate, and 0.05 g manganous sulfate tetrahydrate, per liter, at 37 °C for 18 h.

Glucooligosaccharides-producing strain *Weissella cibaria* YRK005 was isolated from young radish [17]. The strain was also cultured and maintained on MRS agar (BD) at 37 °C for 18 h.

### 2.2. Determination of Antioxidant Activity of Lactic Acid Bacteria

The antioxidant activity of lactic acid bacteria was determined using 2,2-diphenyl-1-picrylhydrazyl (DPPH, Sigma-Aldrich, St. Louis, MO, USA). Two millimolar concentration of DPPH dissolved in methanol was diluted to 0.1 mM before use. Lactic acid bacteria culture grown in MRS was inoculated into MRS broth to achieve a final concentration of 1% (*v/v*) and incubated at 37 °C for 18 h. The culture broth was centrifuged at 21,200× *g* for 5 min to obtain the supernatant, and 10 μL of the supernatant was transferred into a 96-well plate. One hundred and ninety microliters of 0.1 mM DPPH were added and kept in the dark for 30 min at room temperature. The absorbance of the reaction mixture was measured at the wavelength of 517 nm using a spectrophotometer (Epoch microplate reader, Biotek Instruments, Inc., Winooski, VT, USA). A standard curve was prepared with 0–2 mM ascorbic acid. Distilled water was used as a control and 2 mM ascorbic acid was used for positive control. The antioxidant activity was expressed as a DPPH radical scavenging activity and calculated using Equation (1).

DPPH radical scavenging activity (%) = (1 − (A_sample_/A_control_)) × 100
A_sample_: Absorbance of sample
A_control_: Absorbance of control (1)

### 2.3. Determination of Acid Tolerance of Lactic Acid Bacteria

A colony of lactic acid bacteria was inoculated from an agar plate into MRS broth and incubated at 37 °C for 18 h. The culture was inoculated into 4 mL MRS broth to make a final concentration of 1% (*v/v*) and incubated at 37 °C for 18 h. After centrifuging the culture at 21,200× *g* for 2 min, the obtained pellet was washed twice with sterilized saline. The washed cell pellet was resuspended in 4 mL of 100 mM glycine-HCl buffer (pH 2.5) and incubated at 37 °C for 2 h. The cells were spread onto MRS agar plate, incubated at 37 °C for 18 h, and the number of viable cells was counted. The cell pellet without incubation was used as a control. The acid tolerance of lactic acid bacteria was calculated using Equation (2).

Acid tolerance (%) = (Viable cell number of sample/Viable cell number of control)(2)

### 2.4. Determination of Bile Salt Tolerance of Lactic Acid Bacteria

A single colony of lactic acid bacteria was inoculated into MRS broth and incubated at 37 °C for 18 h. Next, the culture was inoculated into MRS with or without 0.3% Bacto oxgall (BD) to make a final concentration of 1% (*v/v*) and incubated at 37 °C for 18 h. The bacterial culture was spread onto MRS agar plate, incubated 37 °C for 18 h, and the viable cell number was counted. The bile salt tolerance was calculated using Equation (3).

Bile salt tolerance (%) = (Viable cell number in the presence of oxgall/Viable cell number in the absence of oxgall) × 100(3)

### 2.5. Adhesion Ability to Intestinal Cells

Caco-2 cells (ATCC^®^ HTB-37^™^) were purchased from American Type Culture Collection (ATCC, Manassas, VA, USA) and cultured in high-glucose Dulbecco’s modified eagle medium (DMEM, Gibco, Grand Island, NY, USA) containing 1% penicillin-streptomycin (Gibco) and 10% fetal bovine serum (CORNING, Corning, NY, USA) at 37 °C in 5% CO_2_ incubator. Lactic acid bacteria were cultured in MRS broth until the number of viable cells was 1 × 10^8^ CFU/mL. Five milliliters of bacterial culture were centrifuged at 15,920× *g* for 1 min, washed three times with Dulbecco’s phosphate-buffered saline (DPBS, Welgene, Gyeongsan, Korea), and resuspended with 1 mL DMEM containing 10% fetal bovine serum (CORNING). Caco-2 cells were seeded in a 24-well plate with 500 μL of DMEM containing 5 × 10^5^ cells per well. The plate was pre-incubated at 37 °C in a 5% CO_2_ incubator for 20 h and washed twice with DMEM containing 10% fetal bovine serum. Two hundred microliters of lactic acid bacteria cells (1 × 10^8^ CFU/mL) and 300 μL of DMEM containing 10% fetal bovine serum were added to Caco-2 cells and incubated at 37 °C in 5% CO_2_ incubator for 2 h. After incubation, the 24-well plate was washed twice with DPBS to remove lactic acid bacteria cells and dead caco-2 cells. Cells attached to the 24-well plate were detached using 200 μL of 0.25% Trypsin-EDTA (Gibco) and collected. Collected cells were washed twice with DPBS at 9420× *g* for 1 min, resuspended in 1 mL of 0.1% Triton X-100 (Sigma-Aldrich), 10-fold serially diluted in saline (3M Company, Maplewood, MN, USA), spread on MRS agar plate, and the viable cell number of lactic acid bacteria were counted after incubation at 37 °C for 18 h. As a control, 200 μL of lactic acid bacteria cells and 800 μL of saline were used. Adhesion ability was calculated using Equation (4).

Adhesion ability (%) = (Adhered bacteria cell number/Initial bacterial cell number) × 100(4)

### 2.6. Identification of Lactic Acid Bacteria

The carbohydrate utilization of lactic acid bacteria was examined using the API 50 CHL kit (API systems, bioMérieux, Marcy-l’Étoile, France) following manufacturer’s instruction and the data were interpreted using Apiweb™ (bioMériuex) available at https://apiweb.biomerieux.com/ (accessed on 10 May 2019).

Lactic acid bacteria genomic DNA was isolated using the Accuprep ^®^ Genomic DNA extraction kit (Bioneer, Daejeon, Korea) following the manufacturer’s instructions. The PCR amplification of the 16S rRNA gene using universal primers 27F and 1492R, nucleotide sequencing of 16S rRNA gene, and phylogenetic tree analysis were carried out as described previously [18,19]. Scanning electron microscopy (SEM) was performed using field-emission SEM (Zeiss SUPRA 66VP, Carl Zeiss Microscopy GmbH, Jena, Germany; installed at the National Instrumentation Center for Environmental Management, Seoul National University, Seoul, Korea).

### 2.7. Production and Purification of Glucooligosaccharides from Weissella cibaria YRK005

Glucooligosaccharides were produced by incubating *W. cibaria* YRK005 in LM broth (2.0 g peptone, 4.0 g yeast extract, 0.01 g NaCl, 2.0 g K_2_HPO_4_, 0.2 g MgSO_4_∙7H_2_O, 0.01 g FeSO_4_∙7H_2_O, 0.01 g MnSO_4_∙7H_2_O, and 0.015 g CaCl_2_∙2H_2_O, per liter) containing 14.7% (*w/v*) sucrose and 13.5% (*w/v*) maltose as described previously [17,20]. Briefly, a colony of *W. cibaria* YRK005 was inoculated into 5 mL MRS broth and incubated anaerobically at 37 °C for 18 h. Three hundred microliters of the culture were inoculated into 30 mL MRS broth and incubated at 37 °C for 9 h. The culture was then inoculated into 2 L LM broth in a 3 L jar fermenter (Fermentec, Cheongju, Korea) and incubated without aeration at 30 °C for 6 h. The pH of the culture was adjusted to 5.0 during the fermentation. The culture was centrifuged at 13,500× *g* for 10 min (Beckman Coulter, Brea, CA, USA) and the supernatant was concentrated under reduced pressure at 60 °C using a rotary evaporator (EYELA, Bohemia, NY, USA).

The concentrated supernatant was loaded onto Bio-gel P2 resin (Bio-Rad Laboratories, Inc., Hercules, CA, USA) in a Glass Econo-Column (1.5 × 120 cm) and the oligosaccharides were separated by gel permeation column chromatography (Bio-Rad Laboratories, Inc.). The flow rate was 30 mL/h and the eluents were fractionated using a fraction collector (Gilson Inc., Middleton, WI, USA). The purified oligosaccharides were freeze-dried (SunilEyela, Seongnam, Korea) and analyzed by thin-layer chromatography (TLC). Silica gel 60 F254 (Merck, Darmstadt, Germany) was used for TLC and the samples were developed twice using 2:5:1.5 nitromethane (Sigma-Aldrich): *n*-propyl alcohol (Sigma-Aldrich):distilled H_2_O. The developed TLC plate was dipped in 0.3%(*w/v*) *N*-(1-naphtyl) ethylenediamine dihydrochloride (Sigma-Aldrich) and 5%(*v/v*) sulfuric acid (Sigma-Aldrich) in methanol (Sigma-Aldrich) and then baked at 121 °C for 5 min [17].

### 2.8. Immunostimulatory Activity of Synbiotics

#### 2.8.1. Application of Synbiotics to RAW 264.7 Cells

Probiotic lactic acid bacteria and glucooligosaccharides were applied to RAW 264.7 macrophage cells, a mouse peritoneal macrophage cell line (TIB-71^TM^, ATCC, Rockville, MD, USA) in two ways to evaluate the immunostimulatory effect of synbiotics on RAW 264.7 cells. One way was the simultaneous treatment of lactic acid bacteria and oligosaccharides in RAW 264.7 cells. Strain SG-030 was grown on RAW 264.7 cells with a multiplicity of infection (MOI, ratio of the number of viable lactic acid bacteria cells to number of viable RAW 264.7 cells) of 2 (1 × 10^6^ CFU bacterial cells/5 × 10^5^ RAW 264.7 cells). For this, 1.5 mL of lactic acid bacteria culture grown in MRS broth was centrifuged at 15,920× *g* for 1 min and the pellet was washed three times with DPBS (Welgene) and resuspended with 500 μL of DMEM containing 10% FBS and 1% penicillin-streptomycin (Gibco). Glucooligosaccharides from *W. cibaria* YRK005 were applied to RAW 264.7 cells at a final concentration of 0.5 μg/mL.

The other way was inoculating lactic acid bacteria culture to 0.1 μg/mL or 0.5 μg/mL oligosaccharides dissolved in modified MRS (mMRS, MRS without glucose) at the concentration of 5% (*v/v*) followed by incubation at 37 °C for 18 h. After incubation, the culture was divided into two preparations. One preparation was the culture broth itself. The other preparation was made by centrifuging the culture broth at 15,920× *g* for 1 min, and the supernatant was filtered with a sterilized PVDF filter (0.22 μm, Woongki Science, Seoul, Korea).

#### 2.8.2. Measurement of Nitric Oxide Production

Nitric oxide (NO) production by RAW 264.7 cells was measured using the Griess reagent. RAW 264.7 cells were attached in a 24-well plate (5 × 10^5^ cells per well) with 500 μL of DMEM. The plates were pre-incubated at 37 °C in a 5% CO_2_ incubator for 20 h. Lactic acid bacteria cells were added to RAW 264.7 cells suspensions to make MOI of 500. Meanwhile, the oligosaccharides were added to RAW 264.7 cells at each 0.1, 1, and 10 mg/mL concentration. Lipopolysaccharides (LPS) at a concentration of 1 μg/mL were used as a control. The plates were cultured at 37 °C for 24 h, and 100 μL of culture supernatant was mixed with 100 μL of Griess reagent (1% sulfanilamide, 0.1% *N*-(1-naphthyl)-ethylenediamine dihydrochloride, and 2.5% phosphoric acid, Sigma-Aldrich) and allowed to react at room temperature and for 15 min. The produced NO was measured using a microplate reader (Epoch, Biotek Instruments, Inc., Winooski, VT, USA) at a wavelength of 450 nm.

#### 2.8.3. cDNA Synthesis and Quantitative Real-Time PCR

Real-time PCR was used to measure inducible nitric oxide synthase (iNOS) gene expression levels and cytokine genes. The treated RAW 264.7 cells were washed with DPBS, and RNA was isolated using easy-BLUE^™^ Total RNA Extraction kit (iNtRON Biotechnology, Inc., Seongnam, Korea) following the manufacturer’s instruction. cDNA was synthesized from the isolated RNA using Transcriptor First Strand cDNA Synthesis Kit (Roche, Basel, Switzerland) according to manufacturer’s instruction.

Real-time PCR was performed by using LightCycler^®^ 96 (Roche) and FastStart Essential DNA Green Master kit (Roche). The sequences of primers (Table S1) and PCR conditions for the analysis of mRNA expression (Table S2) were described in a previous study [21]. In addition, the relative gene expression levels were analyzed using the 2^−ddCT^ method.

#### 2.8.4. Western Blotting

Western blot was conducted to analyze the changes in protein expressions in RAW 264.7 cells treated with synbiotics preparations. RAW 264.7 cells were plated in a 6-well plate (1 × 10^6^ cells/well) and incubated at 37 °C for 20 h. The method for sample treatment for protein extraction was the same as Section 2.8.1. After sample treatment, the cells were incubated at 37 °C for 30 min and washed twice with cold DPBS and treated with 200 μL cell lysis buffer of radioimmunoprecipitation assay (Cell Signaling Technology, Beverly, MA, USA) that containing 1% (*v/v*) phosphatase inhibitor cocktail and 1% (*v/v*) protease inhibitor cocktail (GenDEPOT, Katy, TX, USA). Cells were lysed by reacting on ice for 10 min and centrifuged at 14,000× *g* for 10 min to obtain proteins. The extracted proteins were quantified using Pierce™ BCA protein assay kit (Thermo Fisher Scientific, Waltham, MA, USA).

Western blot analysis was performed using an automated western blot analyzer (JESS, ProteinSimple, San Jose, CA, USA). Total 16 μg of protein solution were separated by 12–230 kDa JESS separation module. After separation, proteins were blocked by antibody diluent followed by incubation with primary antibodies against p38, phospho-p38, extracellular signal-regulated kinases (ERK) 1/2, phospho-ERK 1/2, phosphoinositide 3-kinases (PI3K), phospho-PI3K, c-Jun *N*-terminal kinase (JNK), phospho-JNK, Akt, phospho-Akt (Cell Signaling Technology, Danvers, MA, UA) and anti-rabbit secondary antibodies for 30 min, respectively. The blots were visualized using ECL detection reagent on JESS. The intensity of bands was analyzed by Image J software [22].

#### 2.8.5. Statistical Analysis

The data were obtained by three replicate experiments and expressed as mean ± standard deviation (SD). Experimental data were verified by the student’s *t*-test using SPSS 25 (SPSS Inc., Chicago, IL, USA). Statistical significance between the multi groups was determined by one-way analysis of variance (ANOVA) and Duncan’s multiple range test at the level of *p* < 0.05.

## 3. Results and Discussion

### 3.1. Selection of Lactic Acid Bacteria Which Have Probiotic Properties

The lactic acid bacteria strains with high antioxidant activity, acid tolerance, bile salt tolerance, adhesion to Caco-2 intestinal cells, and NO production were screened among 972 lactic acid bacteria strains isolated in our laboratory to select lactic acid bacteria strains with potential probiotic properties. Among them, 32 strains evaluated by DPPH radical scavenging showed good antioxidant activity (Table S3). Several studies have been reported on the antioxidant activity of probiotics, e.g., ABTS radical scavenging activities of *W. cibaria* D30 (67.81%) and DPPH radical scavenging activities of *Lactobacillus plantarum* C88 (53.05%) [23,24]. Similarly, *L. plantarum* MA2 exhibited resistance to oxidative stress induced by reactive oxygen species by expressing antioxidant enzymes such as super oxide dismutase, glutathione reductase, and NADH peroxidase [25].

Resistances to acid and bile stresses are critical properties for probiotic bacteria. The capability of probiotics to survive, at appropriate levels, in gastrointestinal (GI) tract conditions is important to use in the food industry because the GI tract is a major location that influences the cell viability of lactic acid bacteria [26,27]. In this study, most strains displayed high tolerance to bile salt. However, the tolerance to pH 2.0 and adhesion ability to intestinal cells varied among the strains (Table S3).

NO production in RAW 264.7 macrophage cells treated with lactic acid bacteria, showing high antioxidant activity, acid and bile salt tolerance, and adhesion ability to Caco-2 intestinal cells, were also checked, and some strains showed high NO production. Chang et al. [28] showed that treating viable lactic acid bacteria in RAW 264.7 cells showed a similar level of NO production compared to this study. Kim et al. [29] also reported that NO production of RAW 264.7 cells increased by treatment with *Lactobacillus* sp. The NO production increased dose-dependent in most strains. NO is one of the free radicals produced from L-arginine by NO synthase reaction [30]. When macrophages are activated, NO and cytokine’s production increase and the immune response is promoted [31].

Based on the evaluation of data in Table S3 and Figure S1, strain SG-030, of which antioxidant activity, acid tolerance, bile salt tolerance, adhesion ability to intestinal cells, and NO production were 70.0%, 59.1%, 77.9%, 80.4%, and 24.1 μM, respectively, was finally selected as potential probiotic strain for this study.

### 3.2. Identification of Lactic Acid Bacteria

The selected strain SG-030, isolated from forsythia (*Forsythia koreana* (Rehd.) Naka), was identified by phenotypic, biochemical, and molecular biological analyses. Strain SG-030 was Gram-positive and catalase-negative. When the morphology of strain SG-030 was observed using a scanning electron microscope, its size was determined to be 1.2–1.5 μm in length and 0.4–0.6 μm in diameter (Figure S2a).

When the carbohydrate utilization was analyzed using the API kit, strain SG-030 was able to utilize ribose, galactose, glucose, fructose, mannose, *N*-acetyl glucosamine, amygdalin, arbutin, esculin ferric citrate, salicin, cellobiose, maltose, sucrose, starch, and gentiobiose, which was the same utilization pattern with *L. lactis* KCCM 40,104 as a reference strain (Table S4).

When the nucleotide sequence of 16S rRNA gene, strain SG-030 showed 100% homology with *L. lactis* NCDO 604T, and thereby the strain SG-030 was identified as *L. lactis* by the analysis of phylogenetic tree (Figure S2b). *L. lactis* is useful in the dairy industry because it can ferment lactose and citrate quickly and produce aromatic compounds [32,33].

### 3.3. Cytotoxicity and Immunostimulatory Activity of L. lactis SG-030 and Glucooligosaccharides from Weissella cibaria YRK005

#### 3.3.1. Cytotoxicity of *L. lactis* SG-030 and Glucooligosaccharides from *Weissella cibaria* YRK005 to RAW 264.7 Cells

In Section 3.1, *L. lactis* SG-030 was selected as potential probiotic lactic acid bacteria to use as a probiotic strain in the synbiotics preparation. Glucooligosaccharides from *Weissella cibaria* YRK005 were chosen as prebiotics because of their high prebiotic activity [17]. *Weissella cibaria* YRK005 was isolated from young radish as an oligosaccharides-producing strain in our laboratory. Oligosaccharides were produced by semi-continuous fermentation optimized by Placket-Burman design and response surface methodology and purified using gel permeation column chromatography [17,20]. The purified glucooligosaccharides consisted of 93.8% α-1,6 glycosidic linkages and 6.2% α-1,4 glycosidic linkages, and the molecular weight was determined to be 1.12 × 10^2^ Da with a degree of polymerization of 4–10 [17]. The purified glucooligosaccharides showed prebiotic activity by promoting the growth of probiotic strains such as *Bifidobacterium adolescentis*, *Lactobacillus acidophilus*, *Lactococcus lactis*, and *Lactobacillus pentosus* [17].

**Figure 1:**
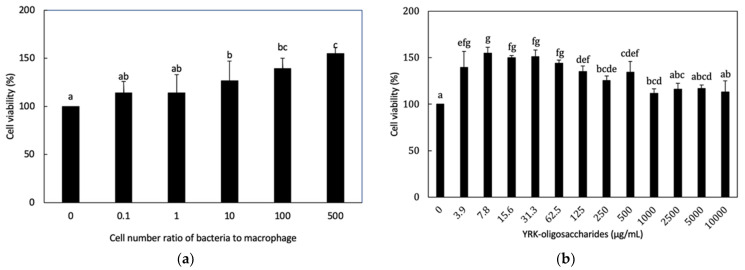
Effects of *L. lactis* SG-030 and oligosaccharides on the cell viability of RAW 264.7 cells. (**a**) Effect of *L. lactis* SG-030 on RAW 264.7 cell viability; (**b**) Effect of oligosaccharides on RAW 264.7 cell viability. Different letters show significant differences among groups at *p* < 0.05 (n ≥ 3). YRK-oligosaccharides: glucooligosaccharides from *W. cibaria* YRK005.

When the cytotoxicities of *L. lactis* SG-030 and the purified glucooligosaccharides on RAW 264.7 cells were examined, *L. lactis* SG-030 and oligosaccharides showed no cytotoxicity in all MOI of bacterial cells or all concentrations of oligosaccharides examined (Figure 1.)

#### 3.3.2. NO Production in RAW 264.7 Cells Treated with Glucooligosaccharides

**Figure 2:**
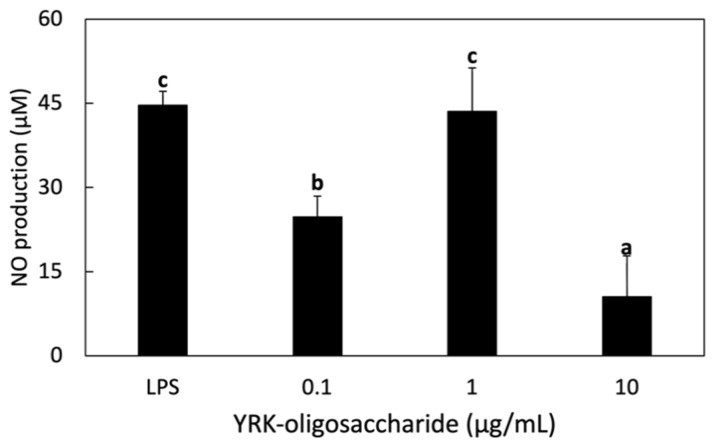
Effect of glucooligosaccharides on NO production in RAW 264.7 cells. RAW 264.7 cells were treated with various concentrations of oligosaccharides for 1 h and then treated with 1 μg/mL LPS for 24 h. NO production was evaluated using the Griess reaction assay. Different letters show significant differences among groups at *p* < 0.05 (n ≥ 3). YRK-oligosaccharides, oligosaccharides from *W. cibaria* YRK005; LPS, lipopolysaccharides.

The immunostimulatory effect of the oligosaccharides produced from *W. cibaria* YRK005 on RAW 264.7 macrophage cells were performed. When 1 μg/mL of LPS was treated, the concentration of NO produced was 44.6 μM, which was similar to that of 1 μg/mL of oligosaccharides. This result showed the high immunostimulatory activity of oligosaccharides on RAW 264.7 cells (Figure 2). Our previous study showed that glucooligosaccharides from *Leuconostoc lactis* CCK940 showed high nitric oxide production and immunostimulatory activity, similar to this study [34,35]. Oligosaccharides from *Leu. lactis* CCK940 are glucooligosaccharides composed of 77.6% α-1,6 and 22.4% α-1,4 glycosidic linkages with the molecular weight of 9.42 × 10^2^ Da [36]. Although the molecular weight of glucooligosaccharides from *Leu. lactis* CCK940 is 9-fold higher than that from *W. cibaria* YRK005, and the composition ratio of α-1,6 and α-1,4 glycosidic linkages are different between two oligosaccharides, immunostimulatory activities of two oligosaccharides were similar. Cheong et al. [37] have shown that treatment with purified glucogalactomannan from *Cordyceps sinensis* at a concentration of 0.1, 0.6, 3, 15 μg/mL increased NO production in a concentration-dependent manner in RAW 264.7 cells, which was also similar to this study.

### 3.4. Immunostimulatory Activity of Synbiotics

#### 3.4.1. Effect of Synbiotics Treatment on NO Production in RAW 264.7 Cells

In this study, synbiotics, the combination of probiotics and prebiotics, were applied to RAW 264.7 cells to evaluate in vitro immunostimulatory activity of synbiotics in two ways. One way was that RAW 264.7 cells were treated with *L. lactis* SG-030 and oligosaccharide simultaneously. The other way was that *L. lactis* SG-030 was cultured with oligosaccharides before treating RAW 264.7 cells. After incubation, the culture broth itself and its supernatant were applied to RAW 264.7 cells. *L. lactis* SG-030 alone and the oligosaccharides alone were also applied to RAW 264.7 cells as controls.

**Figure 3:**
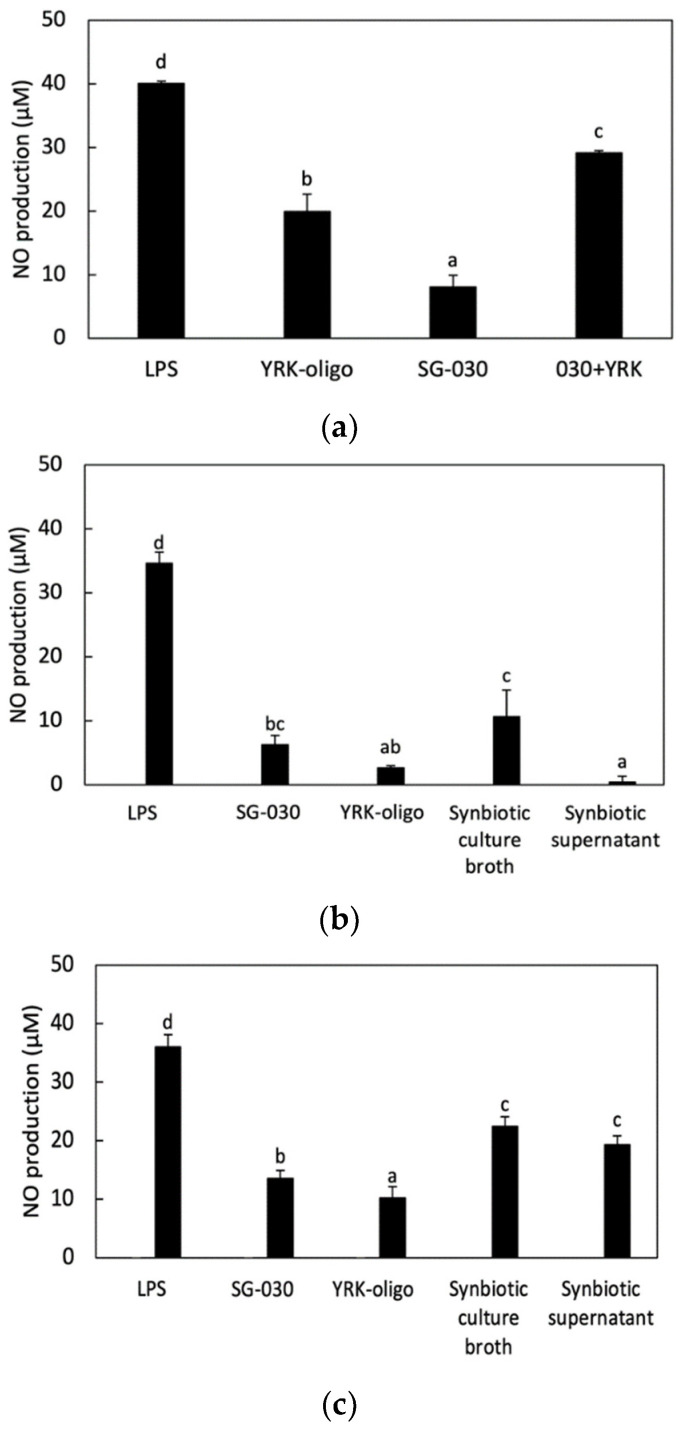
Effects of synbiotics on nitric oxide production in RAW 264.7 cells. (**a**) RAW 264.7 cells were treated with *L. lactis* SG-030 alone, oligosaccharides alone, or synbiotics in the combination of *L. lactis* SG-030 and glucooligosaccharides; (**b**) RAW 264.7 cells were treated with *L. lactis* SG-030 alone, 0.1 μg/mL oligosaccharides alone, synbiotic culture broth, or the supernatant of synbiotics culture; (**c**) RAW 264.7 cells were treated with *L. lactis* SG-030 alone, 0.5 μg/mL oligosaccharides alone, synbiotic culture broth, or the supernatant of synbiotics culture, Different letters show significant differences among groups at *p* < 0.05 (n ≥ 3). LPS, lipopolysaccharides; SG-030, *L. lactis* SG-030; YRK-oligo, 0.1 μg/mL or 0.5 μg/mL of oligosaccharides from *W. cibaria* YRK005; 030 + YRK, combination of *L. lactis* SG-030 and oligosaccharides from *W. cibaria* YRK005.

When RAW 264.7 cells were treated with *L. lactis* SG-030 alone, oligosaccharides alone or synbiotics in the combination of *L. lactis* SG-030 and oligosaccharides, NO production was highest in RAW 264.7 cells treated synbiotics, which showed the synergistic effect of synbiotics (Figure 3A).

When RAW 264.7 cells were treated with *L. lactis* SG-030 alone, 0.1 μg/mL oligosaccharides alone, synbiotic culture broth (with 0.1 μg/mL oligosaccharides and *L. lactis* SG-030), or the supernatant of synbiotics culture (with 0.1 μg/mL oligosaccharides and *L. lactis* SG-030), the treatment with synbiotic culture broth increased NO production the highest among samples (Figure 3B). When RAW 264.7 cells were treated with *L. lactis* SG-030 alone, 0.5 μg/mL oligosaccharides alone, synbiotic culture broth (with 0.5 μg/mL oligosaccharides and *L. lactis* SG-030), or the supernatant of synbiotics culture (with 0.5 μg/mL oligosaccharides and *L. lactis* SG-030), the treatments with synbiotic culture broth and synbiotic supernatant synergistically increased NO production (Figure 3C). In the case of the synbiotic supernatant, the treatment with 0.5 μg/mL oligosaccharides increased NO production higher than the treatment with 0.1 μg/mL oligosaccharides.

The difference between the two methods is that bacteria and oligosaccharides could be taken separately by RAW 264.7 cells in the first method. In contrast, oligosaccharides could be taken by *L. lactis* SG-030 during the culture. Then, the bacteria were supplemented with prebiotics in advance, and oligosaccharides remained in the culture and could be taken by RAW 264.7 cells. Thus, there would be *L. lactis* SG-030 supplemented with prebiotics and oligosaccharides remaining and the metabolites of *L. lactis* SG-030 in the synbiotic culture broth, whereas there would be oligosaccharides remaining used by *L. lactis* SG-030 and the metabolites produced from *L. lactis* SG-030 in the synbiotic culture supernatant.

This result implied that when oligosaccharides were supplemented in a small amount (0.1 μg/mL), there was a small amount of oligosaccharides in the supernatant due to the depletion caused by bacterial uptake. When the oligosaccharides were supplemented in a high amount (0.5 μg/mL), there were enough oligosaccharides to induce NO production. This showed that oligosaccharides had an important role in the synbiotic immunostimulatory activity.

#### 3.4.2. Effect of Synbiotics on Cytokines and INOS Secretions in RAW 264.7 Cells

**Figure 4:**
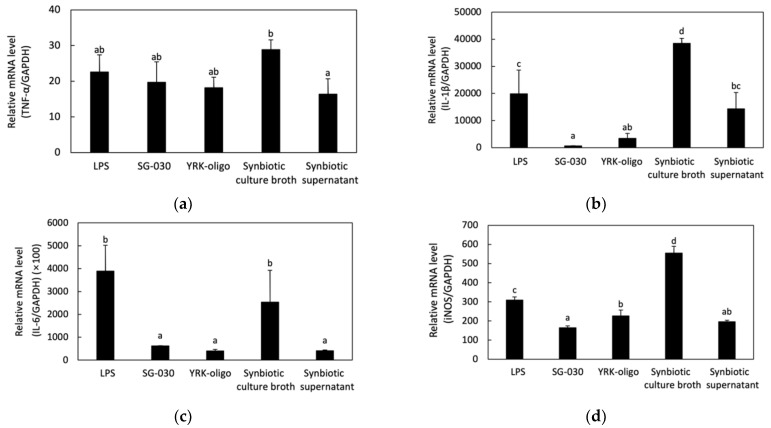
Effects of synbiotics on the mRNA expression of cytokine genes and iNOS gene in RAW 264.7 cells. (**a**) Relative mRNA expression level of TNF-α gene; (**b**) Relative mRNA expression level of IL-1β gene; (**c**) Relative mRNA expression level of IL-6 gene; (**d**) Relative mRNA expression level of iNOS gene. RAW 264.7 cells were treated with *L. lactis* SG-030 alone, oligosaccharides alone, symbiotic culture broth, and symbiotic supernatant. (n ≥ 3). LPS, lipopolysaccharides; SG-030, *L. lactis* SG-030; YRK-oligo, 0.5 μg/mL of oligosaccharides from *W. cibaria* YRK005. Different letters show significant differences among groups at *p* < 0.05 (n ≥ 3).

NO is released along with various pro-inflammatory cytokines such as TNF-α, IL-1β, and IL-6 when macrophages are activated [38]. NO and other pro-inflammatory mediators prevent the invasion of pathogenic microorganisms that cause inflammation [39]. In addition, NO, as an inflammatory mediator, is produced by activation of iNOS, a factor regulated by NF-kB in murine macrophage [40]. Therefore, the immunomodulatory effect of synbiotics on the secretion of pro-inflammatory cytokines or iNOS production in RAW 264.7 cells were evaluated by analyzing the mRNA expression levels of TNF-α, IL-1β, IL-6, and iNOS genes by treatment of synbiotics combined with *L. lactis* SG-030 and oligosaccharides using real-time PCR (Figure 4).

The combination of oligosaccharides and *L. lactis* SG-030, expression levels of TNF-α, IL-1β, and iNOS genes in the cells treated with synbiotic culture broth were higher than the cells treated with LPS, *L. lactis* SG-030 alone, oligosaccharides alone, and synbiotic supernatant. In particular, the expression of IL-1β and IL-6 genes in the cells treated with synbiotic culture broth was significantly higher than that of cells treated with *L. lactis* SG-030 alone, oligosaccharides alone, and synbiotic supernatant. This indicated that synbiotic culture broth had a synergistic effect on the expression of genes involved in immunomodulation (Figure 4).

Hasan et al. [41] reported that the relative gene expression levels of IL-1β and IL-6 increased in organs of olive flounder when fed with a combination of *Bacillus* sp. SJ-10 and β-glucooligosaccharides as synbiotics, indicating the synergistic effect of synbiotics on innate immunity. Hasan et al. [42] also showed that when the combination of *Lactococcus lactis* subsp. *lactis* 12 and β-glucooligosaccharides was fed to olive flounder, their expression levels of TNF-α, IL-1β, and IL-6 increased.

#### 3.4.3. Effect of Synbiotics on the MAPK and PI3K Signaling Pathway in RAW 264.7 Cells

**Figure 5:**
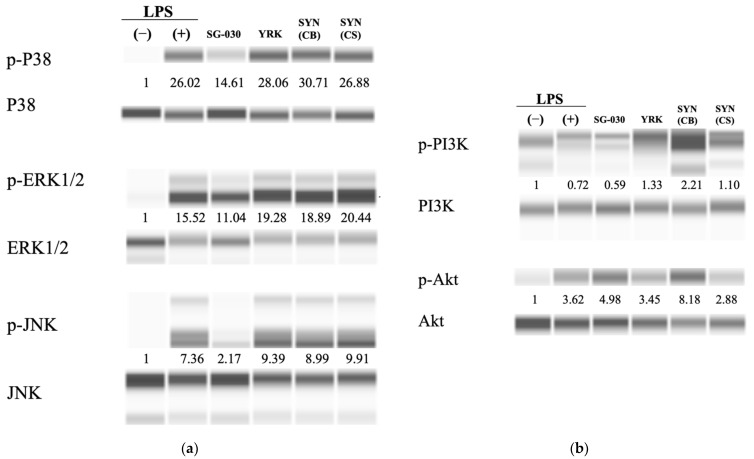
Effects of synbiotics treatment on MAPKs signaling pathways and PI3K/Akt signaling pathways in RAW 264.7 cells. (**a**) Western blot images for the expression of genes in the MAPKs signaling pathways. (**b**) Western blot images for the expression of genes in the PI3K/Akt signaling pathways. One-way ANOVA was used for the comparison of group mean values, followed by Duncan’s multiple range test for assessing the significance of individual comparisons (*p* < 0.05) (n ≥ 3). Different letters represent significant differences among groups. The numbers between blots indicate the relative expression level. SG-030, *L. lactis* SG-030; YRK, 0.5 μg/mL of oligosaccharides from *W. cibaria* YRK005, SYN(CB), synbiotic culture broth; SYN(CS), supernatant of synbiotic culture broth.

The PI3K/Akt signaling pathway regulates macrophage polarization and MAPKs, including ERK, JNK, and P38, regulating gene expression in eukaryotic cells and immune responses such as pro-inflammatory cytokines or affecting cell proliferation. P38, ERK, and JNK require phosphorylation to be activated [43,44,45]. The expressions of P38, ERK1/2, and JNK were investigated by treating RAW 264.7 cells with synbiotics combined with oligosaccharides and *L. lactis* SG-030. The expression level of phosphorylated P38 was higher in cells treated with oligosaccharides, synbiotic culture broth, or synbiotic supernatant than in cells treated with LPS. In particular, it was the highest in the synbiotic culture broth, with a 30.71-fold increase. Likewise, the expression levels of phosphorylated ERK1/2 and JNK were also higher in cells treated with oligosaccharides, synbiotic culture broth, and synbiotic supernatant than in LPS-treated cells, but unlike P38, the expressions of ERK1/2 and JNK were the highest in cells treated with synbiotic supernatant and in cells treated with oligosaccharides, respectively (Figure 5a).

The expression levels of PI3K and Akt also increased with a similar tendency with MAPKs, and were the highest in cells treated with synbiotic culture broth (Figure 5b). These results indicated that synbiotics combined with oligosaccharides and *L. lactis* SG-030 induced phosphorylation of Akt, PI3K, P38, ERK1/2, and JNK, and activated those cytokines. This suggested that the PI3K/Akt and MAPKs pathways were involved in activating macrophages by treatment of synbiotics, leading to secretion of TNF-α, IL-1β, and IL-6, which regulate the immune response [39,46].

**Figure 6:**
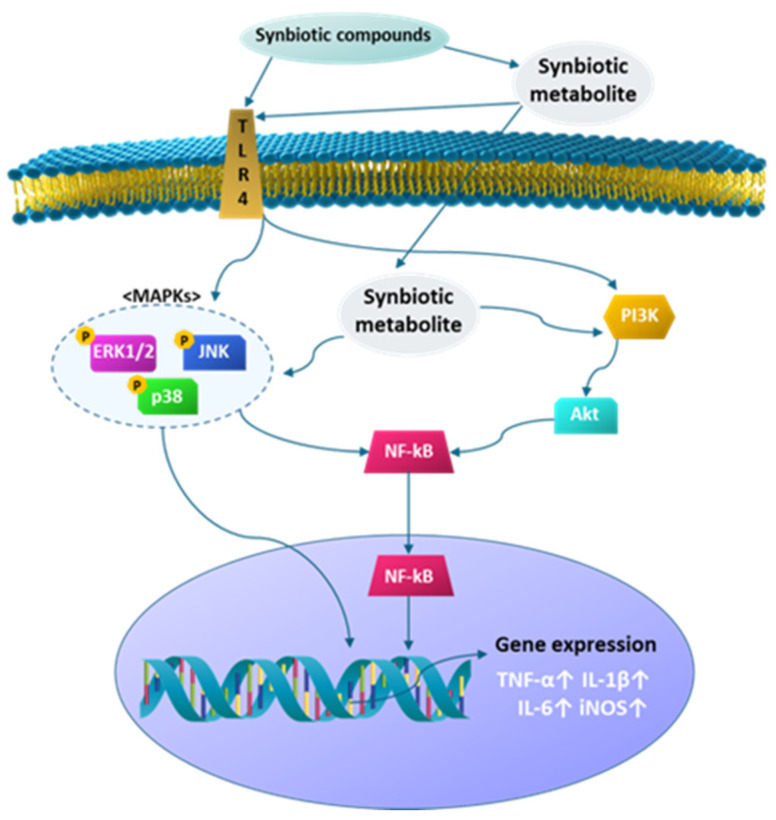
Proposed mechanism for the immunostimulatory activity of synbiotics consisting of glucooligosaccharides and *L. lactis* SG-030 in RAW 264.7 macrophages. TLR4, toll-like receptor 4, NF-κB, nuclear factor kappa-light-chain-enhancer.

The results in this study implied that the treatment synbiotic culture broth induced the expression of IL-6 gene synergistically, and IL-6 activated Janus kinase (JAK) to stimulate PI3K/Akt and ERK1/2 and finally activated the secretion of other cytokines, thereby regulating immune response [38,46,47] (Figure 6).

## 4. Conclusions

The purpose of this study was to prepare synbiotics combined with a probiotic strain and prebiotics and evaluate their immunostimulatory activity using RAW 264.7 macrophage cells. The selection of suitable probiotics and prebiotics is an important criterion for the preparation of synbiotics. To do this, lactic acid bacteria, exhibiting probiotic properties such as high antioxidant activity, acid and bile acid tolerance, adhesion activity to intestinal cells, and NO production in RAW 264.7 macrophage cells, were screened. A lactic acid bacteria strain *L. lactis* SG-030 showed good probiotic properties and was chosen as a probiotic strain, although in vivo study on the probiotic property is needed. In addition, glucodoligosaccharides from *W. cibaria* YRK005 were used as prebiotics due to their growth promotion activity on some probiotic strains.

This study was designed to prepare synbiotics by combining the selected lactic acid bacteria and the purified glucooligosaccharides and confirm whether this combination produces promising synbiotics with immunostimulatory activity. The results showed that synbiotics prepared in this study had synergistic immunostimulatory activity when compared to the results of probiotic strain alone or oligosaccharides alone. Especially, since the treatment of RAW 264.7 cells with synbiotic culture broth induced the expression of cytokine genes and iNOS gene involved in the MAPKs signaling pathways and PI3K/Akt signaling pathways than that with synbiotic culture supernatant, it is necessary to carry out further study to explain this result. Nevertheless, the results in this study confirmed that synbiotics showed a synergistic effect on immunostimulatory activity, suggesting the possibility of the development of synbiotics as a resource for producing beneficial health food.

## Data Availability

Not applicable.

## Supplementary Materials

The following are available online at https://www.mdpi.com/article/10.3390/microorganisms9122437/s1, Figure S1: Effect of lactic acid bacteria on nitric oxide production in RAW 264.7 macrophages, Figure S2: Scanning electron microscopic image and phylogenetic tree of *L. lactis* SG-030, Table S1: Sequence of primers for real-time PCR, Table S2: Condition of real-time PCR, Table S3: Probiotic properties of lactic acid bacteria isolated in our laboratory, Table S4: Carbohydrate utilization of *L. lactis* SG-030 analyzed by API CHL 50 kit.
