# Supplementary material for: Immunostimulatory Activity of Synbiotics Using Lactococcus lactis SG-030 and Glucooligosaccharides from Weissella cibaria YRK005
Source: Microorganisms. 2021 Nov 25;9(12):2437. doi: 10.3390/microorganisms9122437 (PMC8703668; doi:10.3390/microorganisms9122437)
Supplement: Supplementary file 1 [file microorganisms-09-02437-s001.zip › microorganisms-1464264-supplementary.pdf]

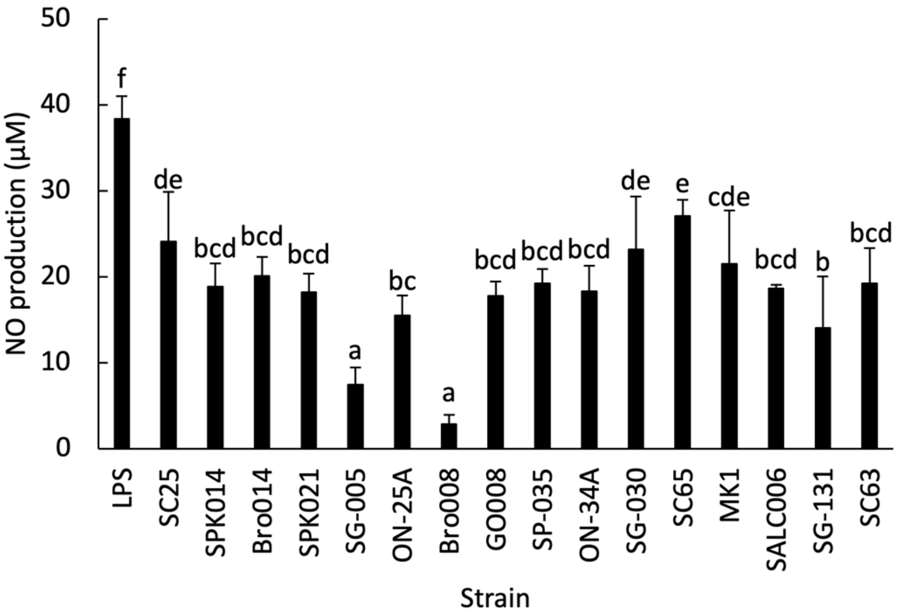

Figure S1. Effect of lactic acid bacteria on nitric oxide production in RAW 264.7 macrophages. The multiplicity of infection (bacterial cells number/macrophage cells number) was 500. Data are expressed as means ± standard deviation of three independent experiments. LPS, RAW 264.7 cells were treated with 1 μg/mL lipopolysaccharides for 24 h.

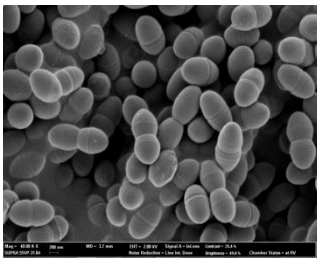

(a)

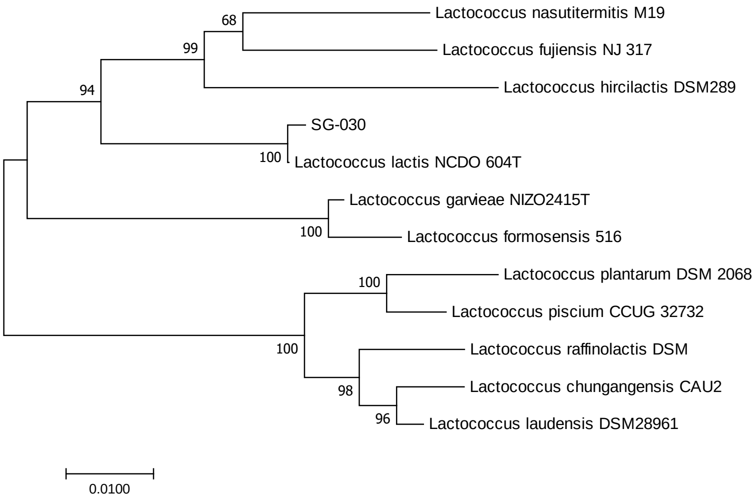

(b)

Figure S2. Scanning electron microscopic image and phylogenetic tree of *L. lactis* SG-030. (a) Scanning electron microscopic image of *L. lactis* SG-030 (×40,000). (b) Phylogenetic tree of *L. lactis* SG-030 generated by neighbor-joining method based on the sequence of 16S rRNA gene.

Table S1. Sequence of primers for real-time PCR.

Gene		Sequence
GAPDH	Forward	5′—ATC CCA TCA CCA TCT TCC AG—3′
	Reverse	5′—CCT GCT TCA CCA CCT TCT TG—3′
TNF-α	Forward	5′—ATG AGC ACA GAA AGC ATG ATC CG—3
	Reverse	5′—CCA AAG TAG ACC TGC CCG GAC TC—3′
IL-1β	Forward	5′—ATG GCA ACT GTT CCT GAA CTC AAC T—3′
	Reverse	5′—CAG GAC AGG TAT AGA TTC TTT CCT T—3′
IL-6	Forward	5′—CAA GAG ACT TCC ATC CAG TTG C—3′
	Reverse	5′—TTG CCG AGT TCT CAA AGT GAC—3′
iNOS	Forward	5′—AAT GGC AAC ATC AGG TCG GCC ATC ACT—3′
	Reverse	5′—GCT GTG TGT CAC AGA AGT CTC GAA CTC—3′

Table S2. Condition of real-time PCR.

Temperature (°C)	Time (s)	Cycle
95	600	1
95	10	
55	30	45
72	15	
95	10	
65	60	1
97	1	

Table S3. Probiotic properties of lactic acid bacteria isolated in our laboratory.

Strain	Isolation source	Antioxidant activity (%)	Acid tolerance (%)	Bile tolerance (%)	Adhesion ability (%)
BKKC329	Kimchi	55.6 ± 9.3	20.6 ± 2.9	87.1 ± 0.6	n.d.
BKKC339	Kimchi	41.4 ± 3.4	20.8 ± 1.4	86.4 ± 0.2	n.d.
Bro008	Broccoli	53.9 ± 4.3	39.7 ± 1.9	98 ± 0.5	53.3 ± 3.7
Bro014	Broccoli	71.9 ± 0.7	14.9 ± 2.1	86.4 ± 1.1	46.7 ± 7.7
GO008	Green onion	57.0 ± 7.4	54.2 ± 1.3	79.2 ± 0.4	70.5 ± 10.2
HW1-1	Green onion	28.2 ± 8.0	n.d.	90.5 ± 2.4	N.D.
MK1	Kimchi	64.2 ± 2.5	57.7 ± 7.4	83.0 ± 1.9	72.2 ± 10.7
ON-25A	Onion	48.3 ± 8.1	49.2 ± 4.1	81.6 ± 0.8	61.6 ± 4.7
ON-34A	Onion	47.8 ± 4.0	60.4 ± 0.0	81.8 ± 1.4	61.4 ± 4.4
PEAR008	Pear	57.8 ± 9.0	n.d.	57.9 ± 3.1	N.D.
SALC006	Salted clam	51.8 ± 1.7	24.4 ± 5.0	83.6 ± 0.5	55.8 ± 11.0
SC10	Salted small octopus	58.8 ± 2.6	n.d.	84.7 ± 2.6	N.D.
SC21	Salted small octopus	55.6 ± 5.7	n.d.	83.6 ± 2.4	N.D.
SC25	Salted small octopus	72.1 ± 5.1	11.8 ± 1.6	89.6 ± 1.6	66.7 ± 7.4
SC5	Salted small octopus	46.9 ± 5.0	n.d.	86.2 ± 2.5	N.D.
SC63	Pickled burdock	68.5 ± 2.4	59.4 ± 1.0	82.8 ± 3.1	75.0 ± 2.6
SC65	Pickled burdock	67.7 ± 2.5	59.8 ± 4.1	90.6 ± 2.4	70.6 ± 11.2
SG-001	Leek	36.3 ± 4.0	7.8 ± 0.5	78.3 ± 1.9	n.d.
SG-005	Potato	48.6 ± 7.3	28.4 ± 6.5	72.3 ± 0.1	50.2 ± 6.6
SG-010	Kimchi	53.1 ± 4.6	n.d.	83.7 ± 3.0	N.D.
SG-025	Flower of Japanese apricot	64.3 ± 9.0	n.d.	88.9 ± 0.2	N.D.
SG-030	*Forsythia koreana* (Rehd.) Nakai	70.0 ± 5.2	59.1 ± 8.6	77.9 ± 0.5	80.4 ± 5.3
SG-131	Chinese cabbage kimchi	65.2 ± 8.9	n.d.	84.1 ± 0.0	N.D.
SG-138	Chinese cabbage kimchi	49.3 ± 3.7	16.3 ± 6.4	70.5 ± 1.7	61.1 ± 5.3
SG-147	Young radish kimchi	56.7 ± 6.2	n.d.	89.7 ± 0.2	N.D.
SG-303	Young radish kimchi	58.6 ± 8.5	n.d.	87.0 ± 0.7	N.D.
SG-309	Chinese cabbage kimchi	56.5 ± 9.1	n.d.	99.0 ± 6.6	N.D.
SG-312	Chinese cabbage kimchi	56.4 ± 1.6	14.8 ± 5.5	87.8 ± 0.1	n.d.
SP-035	Sweet potato	9.12 ± 4.9	57.6 ± 3.3	81.6 ± 0.8	70.6 ± 10.4
SPK014	Sweet pumpkin	62.7 ± 8.6	40.9 ± 3.2	90.1 ± 0.6	54.0 ± 4.3
SPK021	Sweet pumpkin	54.7 ± 5.2	58.8 ± 8.9	82.4 ± 1.2	82.0 ± 10.5
YKC006	Kimchi	43.8 ± 2.0	n.d.	64.5 ± 6.1	N.D.

n.d., not detected; N.D., not determined. Data are expressed as means ± standard deviation of three independent experiments.

Table S4. Carbohydrate utilization of *L. lactis* SG-030 analyzed by API CHL 50 kit.

Carbohydrate	*L. lactis SG-030*	*L. lactis* KCCM 40104	Carbohydrate	*L. lactis SG-030*	*L. lactis* KCCM 40104
Glycerol	-	-	Salicin	+	+
Erythritol	-	-	D-Cellobiose	+	+
D-Arabinose	-	-	D-Maltose	+	+
L-Arabinose	-	-	D-Lactose (bovine origin)	+	-
D-Ribose	+	+	D-Melibiose	-	-
D-Xylose	-	-	D-Saccharose (sucrose)	+	+
L-Xylose	-	-	D-Trehalose	-	+
D-Adonitol	-	-	Inulin	-	-
Methyl-β-D-Xylopyranoside	-	-	D-Melezitose	-	-
D-Galactose	+	+	D-Raffinose	-	-
D-Glucose	+	+	Amidon (starch)	+	+
D-Fructose	+	+	Glycogen	-	-
D-Mannose	+	+	Xylitol	-	-
L-sorbose	+	-	Gentiobiose	+	+
L-Rhamnose	-	-	D-Turanose	-	-
Ducitol	-	-	D-Lyxose	-	-
Inositol	-	-	D-Tagatose	-	-
D-Mannitol	-	-	D-Fucose	-	-
D-Sorbitol	-	-	L-Fucose	-	-
Methyl-α-D-Mannopyranoside	-	-	D-Arabitol	-	-
Methyl-α-D-Glucopyranoside	-	-	L-Arabitol	-	-
N-Acetyl Glucosamine	+	+	Potassium gluconate	-	-
Amygdalin	+	+	Potassium 2-ketogluconate	-	-
Arbutin	+	+	Potassium 5-ketogluconate	-	-
Esculin ferric citrate	+	+			

L. Lactis KCCM 40104 was used as reference strain.
